# Mr. Shorter, on the Vaccine Inoculation

**Published:** 1800-04

**Authors:** John Shorter

**Affiliations:** Surgeon, Bloxham, near Banbury, Oxon


### *To the Editors of the Medical and Phyſical Journal.*

GENTLEMEN,

AS a conſtant reader and well-wiſher of the Medical and Phyſical Journal, I beg leave to tranſmit to you the incloſed letters. If you think they are of ſufficient importance to be made public, do me the favour to give them a place in it as ſoon as you have room. From a peruſal of them, you will perceive I had ſtrong doubts of the efficacy of Cow-pox Inoculation, as a ſubſtitute for Small-Pox, till I received Dr. Jenner's anſwer to the caſes I ſent him.

His gentleman-like letter removed all my doubts, and I have again reſumed the practice.

The facts which have preſented themſelves to my obſervation, tion, hitherto, enable me to ſay, without heſitation, that I am convinced that the Cow-pox Inoculation will ſuperſede the Small-pox. However, as too much evidence cannot be had upon a point of ſo much conſequence to the community, I feel deſirous that every circumſtance relative to the appearance and effects of variolous virus afterwards ſhould be made known; and for theſe reaſons, I trouble you with the letters, having the Doctor's permiſſion to do it. In the country, where Small-pox only now and then makes its appearance, it is a matter of the utmoſt magnitude to have the point at iſſue determined, that individuals may embrace the opportunity of avoiding a moſt dreadful malady, by a ſecure, efficacious, and inoffenſive preventive. I am,

Gentlemen,

Your very humble ſervant,

*Bloxham,*
*Feb.* 17, 1800.

J. SHORTER.

---

### *To Dr.* JENNER.

Sir,

MANY months ſince I peruſed with pleaſure your publication on Cow-pox. The novelty and importance of the ſubject, urged me to inquire farther into it; and I collected ſeveral inſtances that favoured the plan, and others which ſeemed to diſcourage it.

From theſe I ſelected four caſes, two of which very ſtrongly ſubſtantiated your obſervations, and two others which, in my judgment, made equally ſtrong againſt them. Theſe I communicated to Dr. Pearſon in a circumſtantial manner, who very politely anſwered my letter, and cleared up many of my doubts; he alſo kindly ſent me ſome matter on thread, with which I have inoculated ſeveral patients, all of whom had the complaint with no other appearances than on their arms only, excepting in one inſtance, where one of the patients ſhewed me a very fine puſtule with pus in it, on the thigh, exactly reſembling the Small-pox.

Having an opportunity of trying the effect of variolous virus on two perſons who had paſſed through the Cow-pox, I gladly embraced it, thinking it might tend to convince my mind of the great advantages which your tract informed me were to be derived from Cow-pox.

The reſult ſo far exceeds any account that I have noticed, and appears to me ſo neceſſary to be known, that I have taken the liberty of ſubmitting it to your conſideration; and until I am fully ſatisfied, I ſhall deſiſt from proceeding in the practice of inoculating for Cow-pox.

The

The two perſons on whom I made the trial were reſpectable and intelligent, and made ſenſible obſervations on the different appearances of their arms after inoculation. One of them kept an account of himſelf and family from the time of being inoculated with Cow-pox matter, which I ſhall have occaſion to allude to in my relation of their caſes.

William Barrett, Joſeph Barrett, and Ann Barrett, of Adderbury, adults, were inoculated with Cow-pox matter (by Mr. Joſeph Lamb, late a pupil to Mr. Pole, ſurgeon, London) about the middle of laſt June, and went regularly through the complaint. Some time after their recovery, Joſeph expoſed his perſon in the company of a woman with a full crop of Small-pox on her, and received no harm. William was alſo inoculated with variolous matter without effect; however, William ſubmitted to a ſecond trial; and Ann was inoculated at the ſame time. On the 7th of December I inoculated them both with matter on thread, taken only the day before. They ſoon were ſenſible of ſome effect from it. The firſt twenty-four hours they felt ſmarting and itching in the inciſions; and on removing the threads, they obſerved them moiſt. There was little farther to be perceived till the 4th day, when inflammation was evident on William's arm, ſufficient for me to have aſſured him, if he had not had the cow-pox, that I ſhould have been poſitive he had received the infection. Ann's arm appeared very doubtful. From this day to the 7th, the inflammation was progreſſive; and on examining the arms, a puſtule preſented itſelf on Ann's, with matter in it, on the inoculated part; ſhe alſo informed me, that *this day ſhe was very unwell, was feveriſh, and had a tendency to ſickneſs.* William's arm was alſo more inflamed than on the 4th day; the inciſion had a riſing appearance, and felt hard, and towards the lower part there was a puſtule juſt appearing; but he had not the leaſt indiſpoſition this day, or any ſucceeding one. On the 9th day, William called on me, and informed me, that on the 8th he had obſerved matter in the puſtule on his arm; but when I inſpected it this day, it had the uſual aſpect of Small-pox juſt declining: he alſo told me, that the puſtule on Ann's had diſcharged matter on the 8th day; but that on this day, it appeared to be dying, and all indiſpoſition gone.

On the 12th day, I ſaw them both again; Ann's arm had *inflammation, which extended almoſt from the ſhoulder to the elbow,* and ſo painful and ſore that ſhe applied an emollient poultice to it; the inciſion now had a large ſcab on it, and there was on the bend of the arm a ſort of blighted puſtule.

Ruminating on theſe appearances for a moment, I could not but perſuade myſelf ſhe had certainly received the variolous infection, fection, and I frankly told her fo; William's arm alfo retained confiderable inflammation and hardnefs about it, nearly as much as I have obferved when Small-pox puftules have appeared. One thing, however, I muft not omit mentioning, that neither of them were fenfible of the leaft ftiffnefs, fullnefs, or forenefs in the axilla all the whole time; and from this day nothing remarkable occurred.

I have obferved in the courfe of my practice, in feveral inftances, that the Small-pox eruptive fever has come on with vaftly lefs inflammation in the arm; and I am fo perplexed to affign a caufe for fuch unufual appearances after Cow-pox Inoculation, that I muft beg you to fatisfy me before I proceed farther in the practice; and rely on your goodnefs to anfwer the following queftions:

Did the variolous virus affect the fyftem? or was it merely local?

Do you conceive it poffible to have conveyed the Small-pox from the puftule on the inoculated part? or was it poffible that infection could have been conveyed in any manner, even by the clofeft intimacy?

Has any fimilar cafe occurred in your extenfive practice?

Thus far, Sir, I have trefpaffed on your time and patience; but I hope the importance of the fubject will plead my excufe.

I remain, Sir,

Yours, refpectfully,

*Dec.* 25, 1799. JOHN SHORTER, Surgeon,

Bloxham, near Banbury, Oxon.

---

### *To Mr.* SHORTER.

Sir,

I am much obliged to you for your obfervations on the Cow-pox; and am the more pleafed with them, as they convince me you have watched its progrefs with an attentive and fcrupulous eye. However, I muft at once obferve, that the cafes you adduce, *in my opinion*, do not in the leaft militate againft the fafety of the Cow-pox as a preventive of the Small-pox. Pray recollect how feldom we find the fkin infenfible to the action of variolous matter in thofe who have previoufly gone through the Small-pox; the Cow-pox leaves it in the fame ftate. The patients you mention were not infenfible to the local action of the variolous virus; and in one of them, it feems, a very extenfive cuticular inflammation was excited. Now, Sir, allow me to afk, Can you be furprifed (when you confider the fympathetic connection that exifts between the fkin and the ftomach, and confequently the whole conftitution) that an inflammation mation fo extenfive as to reach from the fhoulder to the elbow, fhould occafion fome degree of naufea, and other affections of the body?

The conftitution, you may be affured, gave every reafonable proof of refifting the action of the Small-pox virus completely, the inflammation only occafioned the difturbance; even the lymphatic glands of the axilla were infenfible of its action: but this, indeed, is no teft.

A cafe, fomewhat fimilar to that of the woman you mention, occurred lately in this neighbourhood. A girl, about eighteen years of age, was inoculated with Cow-pox; fhe had the ufual puftule on the arm without feeling the leaft conftitutional fymptom. Some medical people, who knew the hiftory, fuppofed they could, for a certainty, give her the Small-pox by inoculation. She was inoculated, at different periods, in fixteen different points.

Chagrined (very unwarrantably) at the difappointment, a deeper puncture than before was made in one of her arms, I fuppofe through the cutis, fo that the variolous matter came in contact with the cellular membrane. The confequence was dreadful; an inflammation was excited, and fpread to fuch an alarming height, that mortification was expected. The girl, of courfe, was very ill, but no puftules appeared. A boy of my own, who had been inoculated with the Small-pox, and refpecting whofe fafety I had fome doubts, was again inoculated with variolous virus. His arm inflamed very extenfively, and he became unwell. Two years afterwards, he was a third time inoculated, and the fame appearances again took place in his arm, which were fucceeded by boils about his fhoulder. Similar inftances are upon record in great abundance, and others which are ftill more ftriking.

Thefe general obfervations, I prefume, have anfwered your queries fatisfactorily; however, I will with pleafure reply to them in the order in which they ftand.

To the 1ft.—The variolous matter was, doubtlefs, the caufe of the local affection of the arm; which affection difturbed the fyftem. That the conftitution was unfufceptible of its fpecific action, was evinced by the general hiftory of the cafe.

To the 2d.—The matter generated in the arms of your patient was undoubtedly variolous, and would certainly have communicated the Small-pox by inoculation, and probably by its effluvia. This is exemplified by the puftules on Small-pox nurfes, which contain a perfect Small-pox virus. Thefe puftules appear on fome women as often as they expofe themfelves to the difeafe in a malignant ftate. A cafe in illuftration of this fact, is introduced into my fecond Treatife on the Variolæ Vaccinæ.

To

To the 3d.—I don't recollect any case, in my own practice, where the inflammation on the arm became so extensive on the application of the variolous matter after the Cow-pox.

Before I conclude, I must take the liberty of putting one question to you. Are you quite certain that your lancet* did not pass through the cutis, (which in some subjects is very thin) and that the variolous virus did not touch the cellular membrane?

The occurrence of one circumstance that you have communicated to me, I cannot help regretting; which is, the woman being told that she had the Small-pox: for I declare to you, I would not wish for a stronger case of the powers of the Cow-pox, in securing the system from the Small-pox, than those you have laid before me.

I remain,

Your obedient humble servant,

* Having been in the habit of inoculating many years, I presume I must have known it, had my lancet passed through the cutis; the reader will therefore, I trust, believe it did not. J. S.

